# Cardioprotection mediated by exosomes is impaired in the setting of type II diabetes but can be rescued by the use of non‐diabetic exosomes *in vitro*

**DOI:** 10.1111/jcmm.13302

**Published:** 2017-08-25

**Authors:** Sean M. Davidson, Jaime A. Riquelme, Kaloyan Takov, Jose M. Vicencio, Claire Boi‐Doku, Vanessa Khoo, Christian Doreth, Dina Radenkovic, Sergio Lavandero, Derek M. Yellon

**Affiliations:** ^1^ The Hatter Cardiovascular Institute University College London London UK; ^2^ Advanced Center for Chronic Disease (ACCDiS) Facultad de Ciencias Químicas y Farmacéuticas & Facultad de Medicina Universidad de Chile Santiago Chile; ^3^ Cancer Institute University College London London UK

**Keywords:** Exosomes, ischaemia, reperfusion, cardioprotection, diabetes

## Abstract

Many patients with ischaemic heart disease also have diabetes. As myocardial infarction is a major cause of mortality and morbidity in these patients, treatments that increase cell survival in response to ischaemia and reperfusion are needed. Exosomes—nano‐sized, lipid vesicles released from cells—can protect the hearts of non‐diabetic rats. We previously showed that exosomal HSP70 activates a cardioprotective signalling pathway in cardiomyocytes culminating in ERK1/2 and HSP27 phosphorylation. Here, we investigated whether the exosomal cardioprotective pathway remains intact in the setting of type II diabetes. Exosomes were isolated by differential centrifugation from non‐diabetic and type II diabetic patients, from non‐diabetic and Goto Kakizaki type II diabetic rats, and from normoglycaemic and hyperglycaemic endothelial cells. Exosome size and number were not significantly altered by diabetes. CD81 and HSP70 exosome markers were increased in diabetic rat exosomes. However, exosomes from diabetic rats no longer activated the ERK1/2 and HSP27 cardioprotective pathway and were no longer protective in a primary rat cardiomyocytes model of hypoxia and reoxygenation injury. Hyperglycaemic culture conditions were sufficient to impair protection by endothelial exosomes. Importantly, however, exosomes from non‐diabetic rats retained the ability to protect cardiomyocytes from diabetic rats. Exosomes from diabetic plasma have lost the ability to protect cardiomyocytes, but protection can be restored with exosomes from non‐diabetic plasma. These results support the concept that exosomes may be used to protect cardiomyocytes against ischaemia and reperfusion injury, even in the setting of type II diabetes.

## Introduction

Myocardial ischaemia and reperfusion (IR) injury are a major cause of cell death in patients who experience a myocardial infarction. As the majority of myocytes are terminally differentiated and unable to proliferate, treatments that increase cell survival after infarction would be of great clinical benefit. However, many patients with ischaemic heart disease also have diabetes. In the USA, the occurrence of diabetes in patients undergoing isolated primary coronary artery bypass grafting increased from 33% to 40% between 2000 and 2009 1. The presence of diabetes can impair cardioprotective strategies and worsen outcome 2, 3. For example, the incidence of death or re‐infarction after acute coronary syndromes is significantly higher in patients who have diabetes compared with those who do not 4. This illustrates the need for cardioprotective treatments that are effective in the setting of diabetes.

Exosomes are nano‐sized, lipid bilayer vesicles able to communicate signals between cells 5, 6, 7, 8. They are defined as ~100 nm diameter derivatives of the endosomal compartment 7. We previously showed that plasma exosomes can protect the hearts of rats from IR injury, both *in vitro* and *in vivo* 9. Furthermore, exosomes directly protected primary cardiomyocytes against injury in an experimental model simulating IR *in vitro* 9. The mechanism of protection involved HSP70 on the exosomal surface, which stimulated toll‐like receptor 4 (TLR4) receptors on cardiomyocytes. This led to the activation of ERK1/2 and p38 MAPK, kinases previously identified as part of the reperfusion injury salvage kinase (RISK) pathway of cardioprotection 10, 11. P38 MAPK subsequently phosphorylated HSP27, a member of the highly cytoprotective family of small heat shock proteins 9. As clinical trials commence using exosomes in a variety of disease settings (primarily cancer to date, but with other fields progressing rapidly 7), it is vital to understand the biological capabilities of exosomes and their signalling pathways.

Here, in order to further our investigations into exosomes as potential treatments for myocardial IR injury, we investigated plasma exosomes from Goto Kakizaki (GK) rats with type II diabetes and found they have lost the ability to active cardioprotective signalling pathways. Although exosomes from non‐diabetic rats or humans reduced cell death in cardiomyocytes subject to hypoxia and reoxygenation, those from diabetic individuals were no longer able to do so. Plasma exosomes originate from various sources 12 including endothelium, muscle, platelets, leucocytes and erythrocytes. We isolated exosomes from primary endothelial cells (HUVECs) and showed they can protect cardiomyocytes against hypoxia and reoxygenation. Although hyperglycaemic culture did not alter rates of exosome production or exosomal morphology, it eliminated their cardioprotective ability. However, we found no evidence that hyperglycaemia alters exosomal HSP70 levels, or that glycation of HSP70 renders it incapable of inducing protection. Lastly, in order to investigate their potential for therapeutic application, we investigated whether exosomes from non‐diabetic individuals are able to protect cells from diabetic hearts.

## Materials and methods

All human and animal studies were approved by the appropriate institutional review boards. Animals were treated in accordance with the Animals (Scientific Procedures) act 1986 published by the UK Home Office and the Guide for the Care and Use of Laboratory Animals published by the US National Institutes of Health (revised 2011). The study of human samples was performed according to ethics approval reference 13/LO/0222 and Declaration of Helsinki principles.

### Animals

Male GK or Wistar rats (aged circa 4 months, 250–300 g) were obtained from Charles River, United Kingdom. Animals were treated in accordance with the Animals (Scientific Procedures) act 1986 published by the UK. Home Office and the Guide for the Care and Use of Laboratory Animals published by the US National Institutes of Health (Publication No. 85‐23, revised 1996).

### Primary cardiomyocyte isolation and hypoxia‐reoxygenation studies

Adult rats were anaesthetized with 200 mg/kg i.p. Na‐pentobarbital, the hearts were isolated and perfused *via* the aorta. Cardiomyocytes were isolated by collagenase II perfusion using a standard method and plated on laminin in 6‐well tissue‐culture plates 11. Cells were subject to hypoxia and reoxygenation with a buffer simulating the ischaemic milieu containing 128 mM NaCl, 2.2 mM NaHCO_3_, 14.8 mM KCl, 1.2 mM MgSO_4_, 1.2 mM K_2_HPO_4_, 1 mM CaCl_2_, 10 mM Na‐lactate (pH 6.4) in a hypoxic chamber containing 95% N_2_/ 5% CO_2_ for 3 hrs, after which cells were placed in a standard incubator in normal medium for 1 hr. The percentage cell death was determined by staining dead cells with propidium iodide and counting cells in three separate fields per well (*i.e*. 100–150 cells per group per experiment). Control cells were incubated in normoxic medium 118 mM NaCl, 22 mM NaHCO_3_, 2.6 mM KCl, 1.2 mM MgSO_4_, 1.2 mM K_2_HPO_4_, 1 mM CaCl_2_, 10 mM glucose (pH 7.4 gassed with 95% O_2_ / 5% CO_2_). Cardiomyocytes were treated with 10^8^/ml (0.1 μg) plasma exosomes or 1 ng/ml endotoxin‐free recombinant HSP70 (Enzo Life Sciences, Exeter, UK), and incubated for 30 min. prior to ischaemia. Samples of HSP70 were pre‐incubated for 7 days, 37°C in 20 mM glucose, mannitol or methylglyoxal. TAK‐242 was used at 5 μM. As a positive control, the cardioprotective agent insulin (100 nM) was added at reoxygenation.

### Rat exosome preparation

Rats were anaesthetized with 200 mg/kg Na‐pentobarbital i.p. and placed on a heated mat. After thoracotomy, 4 ml blood was rapidly removed into a citrated vaccutainer™ to minimize platelet activation. Exosomes were prepared by standard differential centrifugation as follows: centrifugation at 1600 × *g* 20 min. RT to obtain plasma, then at 10,000 × *g* 30 min. RT to remove cells and platelets, then twice at 100,000 × *g* 60 min., 4°C with an MLA‐55 rotor, washing with Ca^2+^ and Mg^2+^ free PBS 9, 13. The vehicle control consisted of PBS alone.

### Human exosome preparation

After written consent, exosomes were isolated from patients scheduled to undergo cardiac bypass surgery, aged 50–84 years, with or without Type II diabetes. Sixty microliters blood was drawn from the antecubital vein by butterfly syringe into citrated vaccutainers™ (BD Biosciences, Oxford, UK). The first two vaccutainers were excluded from the exosome isolation procedure to avoid artefacts due to platelet activation. The blood was centrifuged 20 min. at 1600 × *g*, RT, to obtain plasma, which was then centrifuged 30 min. at 10,000 × *g*, RT to remove platelets. The supernatant was ultracentrifuged three times (for 90, 60, then 60 min.) at 100,000 × *g*, 4°C, and washed with Ca^2+^ and Mg^2+^ free PBS, to pellet exosomes 9, 13.

### Cell culture

HUVEC‐pooled cells were obtained from Lonza and cultured in EGM‐2 BulletKit (Lonza, Slough, UK) for up to 20 population doublings, according to the manufacturer's instructions. To prepare exosomes from cultured endothelial cells, medium was washed and replaced with medium containing FCS that had been pre‐cleared of exosomes by ultracentrifugation for 20 hrs at 100,000 *g*, 4°C. The absence of exosomes was verified by NTA. For hyperglycaemic culture, 14.5 mM glucose was added to bring the total concentration to 20 mM. To control for effects due to molarity, 14.5 mM mannitol was added to separately cultured cells. Cells were cultured for 24 hrs before harvesting the medium and isolating exosomes using the ultracentrifugation method described above for rat plasma. Cardiomyocytes were treated with 10^7^/ml HUVEC exosomes for 30 min. before exposure to hypoxia and reoxygenation.

### Electron microscopy

Electron microscopy was performed on a Joel 1010 transmission electron microscope (Joel Ltd, Warwickshire, UK) after a standard staining procedure with 0.5% uranyl acetate 13. Diameter was calculated from 5 to 15 images containing 100–200 vesicles.

### Nanoparticle tracking analysis

Concentration and size of exosomes were determined the using a Nanosight LM10‐HS (Nanosight Ltd, Malvern, UK) as described 9 with constant flow injection. The same dilution was used for all the samples for consistency. Three recordings of 30 sec. each were captured and analysed, and the data from at least 1500–3000 individual particle tracks were analysed per sample.

### DELFIA protein quantification

Exosome proteins were quantified using a previously validated dissociation‐enhanced lanthanide fluorescence immunoassay (DELFIA) 14. Briefly, 5–10 μl of each sample was diluted to 100 μl in PBS, added to high‐binding ELISA plates, and then incubated overnight at 4°C. The plates were washed three times with DELFIA wash buffer (PerkinElmer, Cambridge, UK). Wells were blocked with 100 μl 1% BSA in PBS for 1 hr at room temperature, and then washed three times. Primary antibodies (CD81 clone JS‐81; HSP70 clone N27F3‐4) were added at 1 μg/ml and plates incubated 2 hrs at room temperature. After washing three times, goat anti‐rabbit IgG or goat anti‐mouse IgG1 was added (1:2000 in blocking buffer), and incubated 1 hr at room temperature. Plates were washed three times, and 1:1000 streptavidin–Europium conjugate in DELFIA Assay Buffer (PerkinElmer) was added and incubated 1 hr. Finally, after six washes, 100 μl of the DELFIA Enhancement Solution was added, and the plate was shaken 2 × 5 min. on the plate reader. Time‐resolved fluorimetry was performed using a Pherastar plate reader (BMG Labtech, Aylesbury, UK), with excitation of 337 nm, detection at 620 nm, integration time set at 200 μs and lag time of 60 μs.

### Flow cytometry

Expression of surface marker molecules was measured using a standard protocol in which exosomes were bound to aldehyde microspheres of 4 μm with some modifications 13. Briefly, macromolecular IgG complexes were cleared from 2 × 10^9^ exosomes (quantified by NTA) by an immunoprecipitation pre‐clearing protocol using G protein sepharose (Protein G sepharose; GE Healthcare, Chicago, Illinois, USA). Exosomes were then bound to 5 μl of aldehyde/sulphate latex microspheres (4% w/v, 4 μm Molecular Probes, Thermofisher Scientific, Waltham, MA USA) (a ratio of ~330 exosomes per microsphere), in 1 ml of sterile PBS with agitation at 4°C. Exosome‐coated microspheres were blocked with 3% fatty acid‐free BSA for 1 hr at 4°C, then incubated with isotype control or specific primary antibody against HSP70, cmHsp70.1‐FITC 15. For the analysis of cells or microspheres, we used singlet‐gated events on SSC/pulse‐width. All samples were analysed using an Accuri C6 flow cytometer (BD Biosciences).

### Western blot analyses

For signalling experiments, 10^8^ exosomes/ml were added to cardiomyocyte cultures acutely for 5 min., as indicated, then the cells were rapidly washed in cold PBS and lysed on ice in a buffer containing 100 mM NaCl, 10 mM Tris (pH 7.6), 1 mM EDTA (pH 8.0), 2 mM sodium pyrophosphate, 2 mM sodium fluoride, 2 mM b‐glycerophosphate and a protease inhibitor cocktail. Where indicated, exosomes were pre‐incubated with antibodies (cmHsp70.1) for 30 min. at a ratio of 1 μg antibody per 5 μg exosomes. 100 nM insulin was used as a positive control for Akt phosphorylation. The protein concentration of cell lysates was determined by BCA assay (Pierce, Thermofisher Scientific, Waltham, MA USA) and after addition of 20 μg protein per well separated on a 10% SDS‐PAGE gel. Proteins were transferred to nitrocellulose membranes and were probed using primary antibodies from Abcam (Milton, Cambridge, UK): antibody 3C to argpyrimidine; Cell Signalling Technologies (Boston, MA, USA): ERK1/2 (#9102), Phospho‐ERK1/2 (Thr202/Tyr204) (#9101), Akt (#9272), Phospho‐Akt (Ser473) (#9271), phospho Hsp27 Ser‐82(#12594); and Santa Cruz Biotechnology (Dallas, Texas, USA): beta‐actin (#47778); followed by fluorescent secondary antibodies, and detection using the Li‐cor Odyssey at an intensity set to avoid any saturation of bands. The ratio of phosphorylated Erk1/2 to total Erk1/2 was calculated, and phospho‐HSP27 was measured relative to beta‐actin as a loading control.

### Statistical analysis

Data are shown as mean ± S.E.M. Pairwise comparisons were made by Student's *t*‐test. One‐way anova was followed by post‐test analysis using the Tukey test for multiple comparisons. Repeated measures anova was used when replicate cell cultures or primary cell isolates were treated under each of the conditions, followed by Tukey test. Exosomes size distributions were compared using a Mann–Whitney *U*‐test or Kruskal–Wallis test for 2 or 3 groups respectively. *P* < 0.05 was considered significant. Degrees of significance were indicated as follows: *P < 0.05, **P < 0.01, ***P < 0.001.

## Results

Exosomes were isolated from normoglycaemic rats (fasting blood glucose 5–6 mM) or from hyperglycaemic GK rats with type II diabetes (fasting blood glucose 8–12 mM). Exosomes were also isolated from patients undergoing cardiac surgery who were either non‐diabetic or who were known to have established type II diabetes. Exosomes were also isolated from HUVECs cultured in hyperglycaemic culture conditions (20 mM glucose) or normoglycaemic conditions (5.5 mM glucose with addition of 14.5 mM mannitol to control for osmolarity).

**Figure 1:**
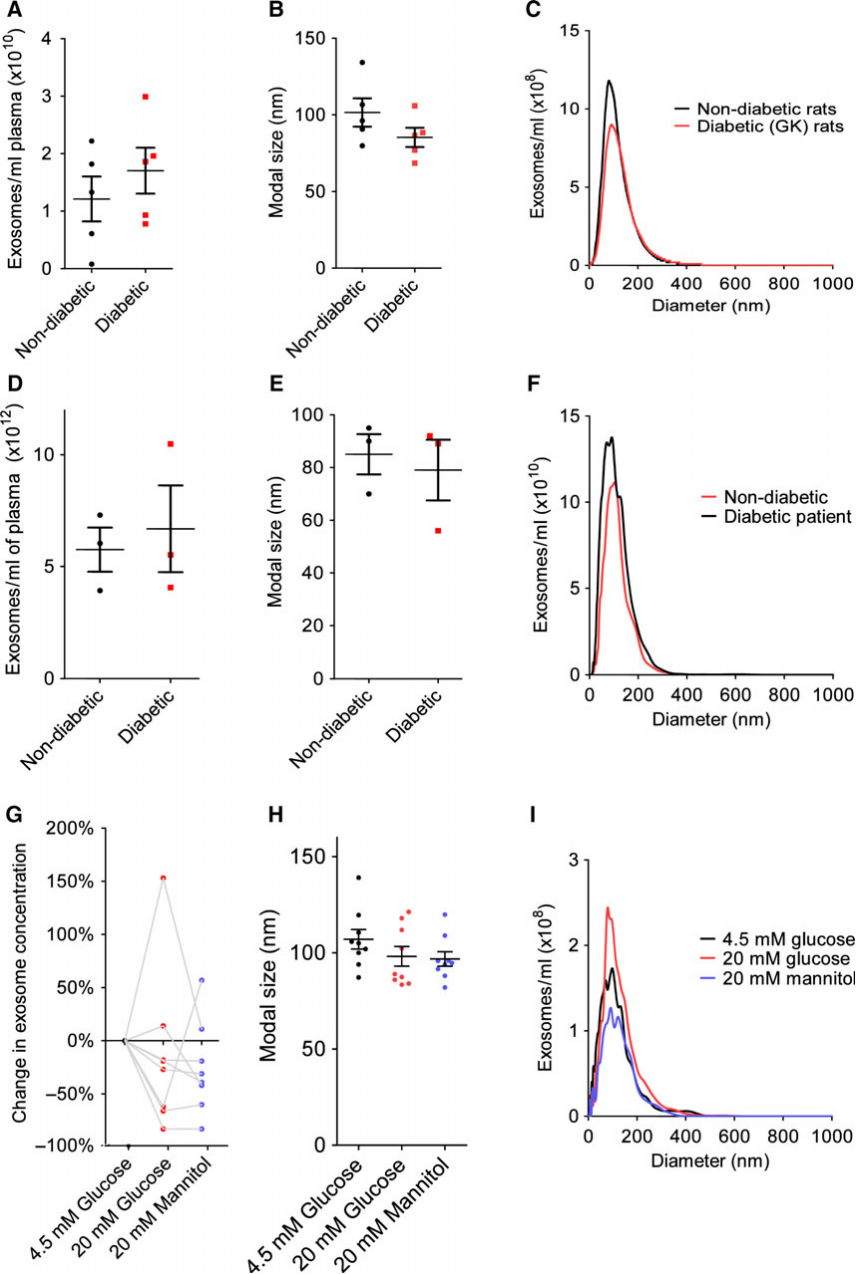
The concentration and modal size of exosome preparations as determined by Nanoparticle tracking analysis. The concentration (**A**) and size (**B** and **C**) of exosomes isolated from non‐diabetic rats and GK diabetic rats. *N* = 5 per group. The concentration (**D**) and size (**E** and **F**) of exosomes isolated from patients with or without type 2 diabetes. *N* = 3 per group. The effect of hyperglycaemic conditions (20 mM Glucose), or iso‐osmotic control conditions (20 mM Mannitol), compared to normoglycaemic conditions (4.5 mM Glucose), on the concentration of exosomes isolated from HUVECs (**G**), and their average size (**H** and **I**). *N* = 8 per group. Statistical comparison was by *t*‐test (**A** and **D**), or repeated measures anova performed on the raw concentration values (**G**).

Exosomes were quantified and characterized by nanoparticle tracking analysis (NTA) (Fig. 1A–I). The concentration of exosomes was 1.2 ± 0.4 × 10^10^/ml in non‐diabetic rats and was not significantly different in diabetic rats (*P* = 0.4, *N* = 5; Fig. 1A). There was no difference in protein quantity per exosome between non‐diabetic and diabetic rat samples (Fig. S1). The concentration of exosomes was 5.8 ± 1.0 × 10^12^/ml in non‐diabetic humans and was not significantly different in diabetic humans (*P* = 0.7, *N* = 3; Fig. 1D). The concentration of exosomes isolated from normoglycaemic HUVECs was 8.4 ± 1.5 × 10^9^/ml. The concentration of exosomes in replicate dishes cultured in hyperglycaemic conditions, or under iso‐osmotic control conditions was not significantly different (*P* = 0.3, *N* = 8; Fig. 1G). The modal size of isolated vesicles was ~100 nm as expected for a population consisting predominantly of exosomes and was not significantly different between diabetic and non‐diabetic rats or humans, or between normoglycaemic and hyperglycaemic cell cultures (Fig. 1B, E, H).

**Figure 2:**
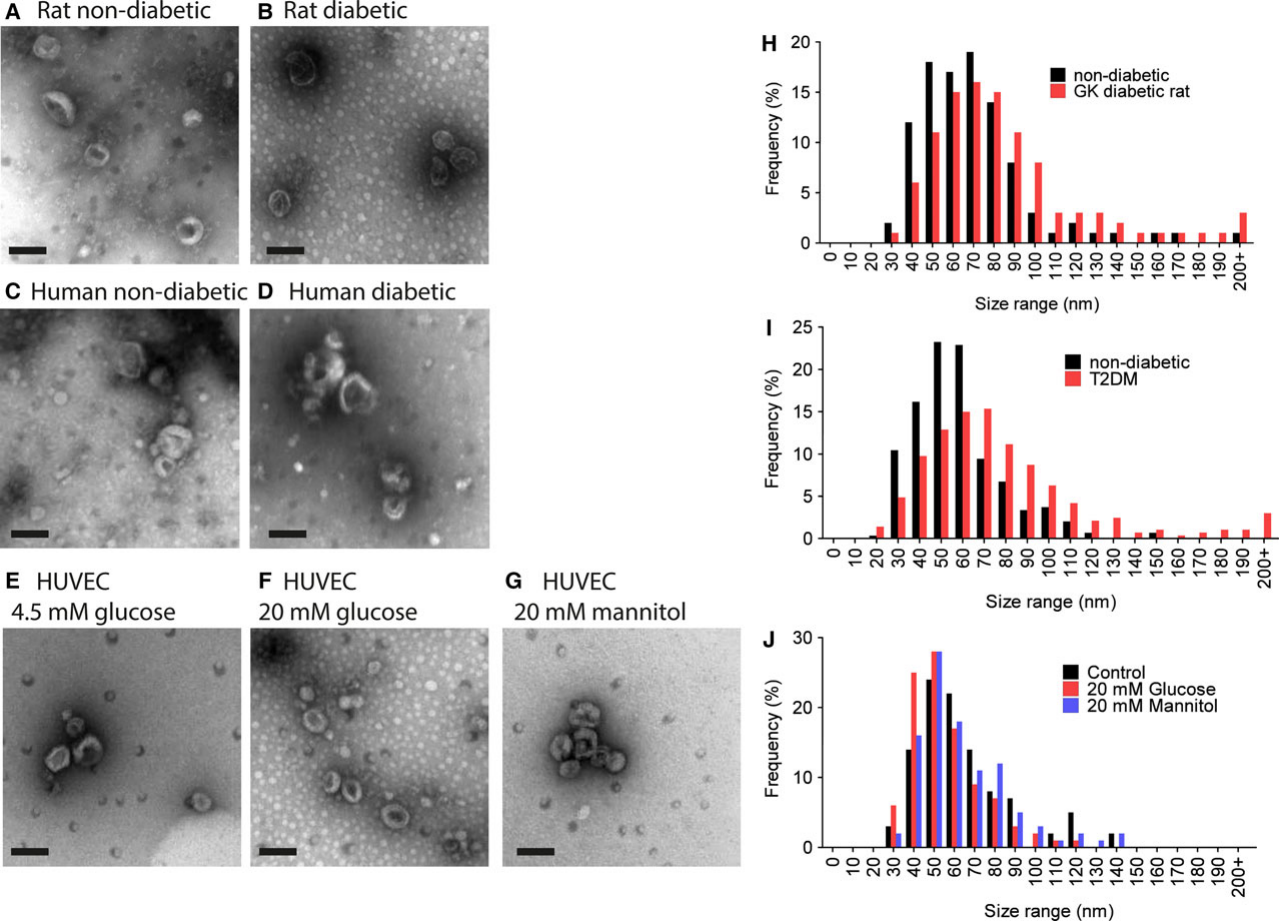
Transmission electron microscopy (TEM) of exosomes isolated from the blood of rats (**A** and **B**), human patients (**C** and **D**) or from cultured HUVECs under the cultured conditions indicated (**E**–**G**). Bar = 100 nm. (**H**–**J**) Size distribution diameters from TEM images of vesicles from rats (**H**) (*N* = 399 and 505), humans (**I**) (*N* = 297 and 284) and HUVECs (**J**) (*N* = 59, 294 and 131). No significant difference was found when distributions were compared by Mann–Whitney *U*‐test or Kruskal–Wallis test for 2 or 3 groups, respectively.

The identity of the isolated vesicles as exosomes was confirmed by transmission electron microscopy. Exosomes isolated from each of the conditions appeared similar in terms of morphology and size (Fig. 2A–G). Histograms of exosome size distribution (Fig. 2H–J) suggested there might be a slight increase in the size of exosomes from diabetic rats and humans, but this was found not to be significant.

**Figure 3:**
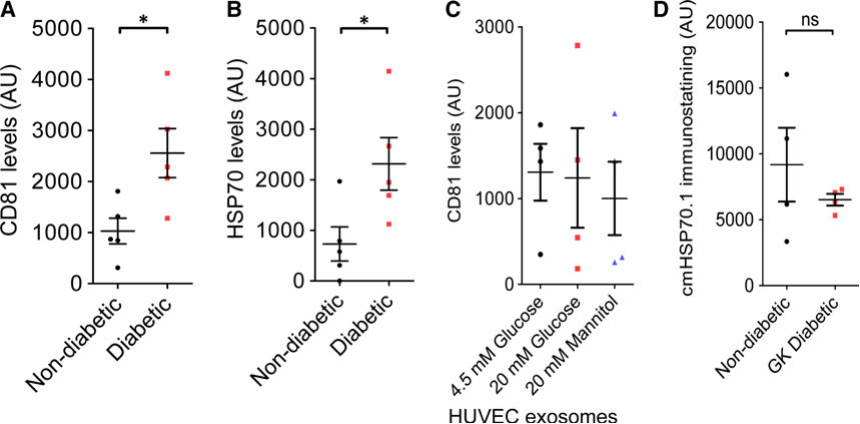
Content of exosome marker proteins CD81 and HSP70 in exosomes isolated from rats (**A** and **B**), or HUVECs (**C**). Markers were measured using an ELISA‐based ‘DELFIA’ assay (see methods). *N* = 5 (**A** and **B**) or 4 (**C**). *P < 0.05. (**D**) Quantitative immunostaining of levels of the cmHSP70.1 epitope of HSP70 determined by a flow cytometry‐based assay of exosomes isolated from non‐diabetic rats and GK rats (*N* = 4). Statistical comparison was by *t*‐test (**A**,** B**,** D**), or anova (**C**).

The identity of the exosomes was confirmed by measuring the presence of exosome markers CD81, a tetraspanin, and HSP70, using an ELISA‐based assay. HSP70 protein is present in exosomal surface membrane and is commonly used as a marker of exosomes. A significant increase in CD81 and HSP70 levels measured in exosomes isolated from diabetic rats (Fig. 3A and B). HUVEC exosomes contained CD81 (Fig. 3C), although interestingly HSP70 was undetectable in HUVEC exosomes. The levels of CD81 were unchanged by exposure to high glucose or mannitol (Fig. 3C).

We previously used a neutralizing antibody to HSP70 (cmHsp70.1) to demonstrate that HSP70 localized on the surface of exosomes induces cardioprotection by signalling to TLR4 9. Using a flow cytometry‐based assay we found that surface levels of the protective HSP70 epitope were not significantly different between exosomes isolated from non‐diabetic and diabetic rats (Fig. 3D).

**Figure 4:**
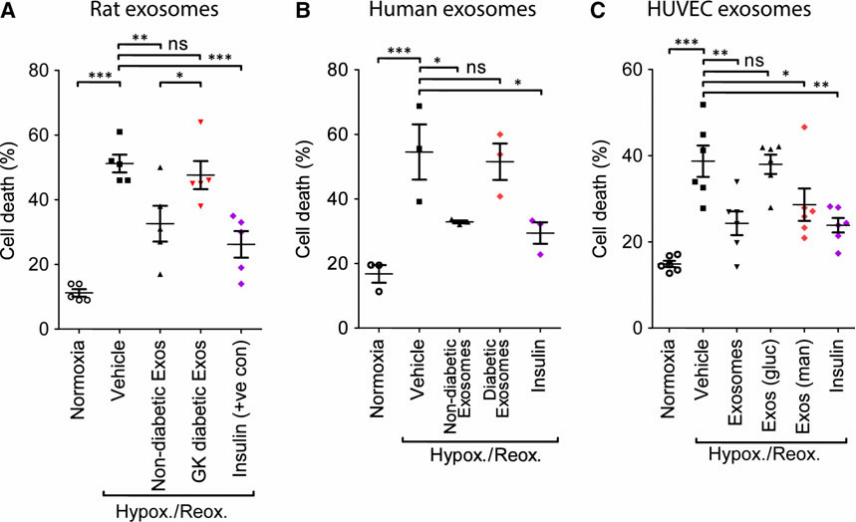
Exosomes from diabetic rats or humans or from endothelial cells cultured under hyperglycaemic conditions were unable to protect primary adult cardiomyocytes from death after hypoxia and reoxygenation. Cardiomyocytes were exposed to 10^8^/ml exosomes from non‐diabetic or GK rats (**A**), non‐diabetic or diabetic patients, (**B**), or 10^7^/ml exosomes from HUVECs cultured in 20 mM glucose (gluc) or 20 mM mannitol (man). (**C**). The cardioprotective agent, insulin was used as a positive control in each case. Cell death was assessed by propidium iodide staining. *N* = 5(**A**), 3(**B**) or 6 (**C**) independent experiments. Statistical comparison was by repeated measures anova. *P < 0.05; **P < 0.01; ***P < 0.001; ns = non‐significant in the comparisons indicated.

To compare cardioprotection by exosomes from normoglycaemic and diabetic samples, a cell‐based model simulating IR was used. Primary adult rat cardiomyocytes were isolated and pre‐treated for 30 min. with 10^8^/ml exosomes (determined by NTA) isolated from non‐diabetic or diabetic rats, before exposure to 3 hrs hypoxia and 1 hr reoxygenation. In normoxic cell cultures, cell death varied between 10% and 20% (Fig. 4A–C, Fig. S2–S4). Hypoxia and reoxygenation increased cell death significantly to 51 ± 3% (*P* < 0.001; Fig. 4A). Insulin was used as a positive control because it provides strong, reproducible protection in this model 11 and was shown to reduce cell death significantly to 26 ± 4% (*P* < 0.001; Fig. 4A). Exosomes from non‐diabetic rats were also cardioprotective in this model, as expected, and reduced cell death to 33 ± 5% (*P* < 0.01) (Fig. 4A). In contrast, exosomes isolated from GK rats were not cardioprotective, and resulted in 48 ± 4% cell death after hypoxia and reoxygenation (*N* = 5; Fig. 4A).

In separate experiments, exosomes isolated from patients with type II diabetes were also unable to protect primary cardiomyocytes cells from hypoxia and reoxygenation injury (cell death 50 ± 8%, despite exosomes from non‐diabetic human blood reducing cell death from 54 ± 9% to 29 ± 2% (*P* < 0.05; Fig. 4B), in line with previous results 9.

We investigated whether the exosomes produced by endothelial cells were equally as protective in this model. Exposure of cardiomyocytes to exosomes produced by HUVECs reduced cell death caused by hypoxia and reoxygenation from 39 ± 4% to 24 ± 3% (*P* < 0.001; Fig. 4C). Diabetes is a complex disease which typically involves hyperinsulinaemia and dyslipidaemia in addition to the diagnostic characteristic of hyperglycaemia. In order to investigate whether exposure to hyperglycaemia was sufficient to eliminate protection by exosomes, we cultured HUVECs in medium containing 20 mM glucose, or an iso‐osmotic control using mannitol. Mannitol did not adversely affect protection afforded by exosomes, resulting in 29 ± 4% cell death (*P* < 0.001 *versus* vehicle). In contrast, exosomes from cells in hyperglycaemic conditions were not protective (39 ± 2% cell death, *N* = 6; Fig. 4C).

**Figure 5:**
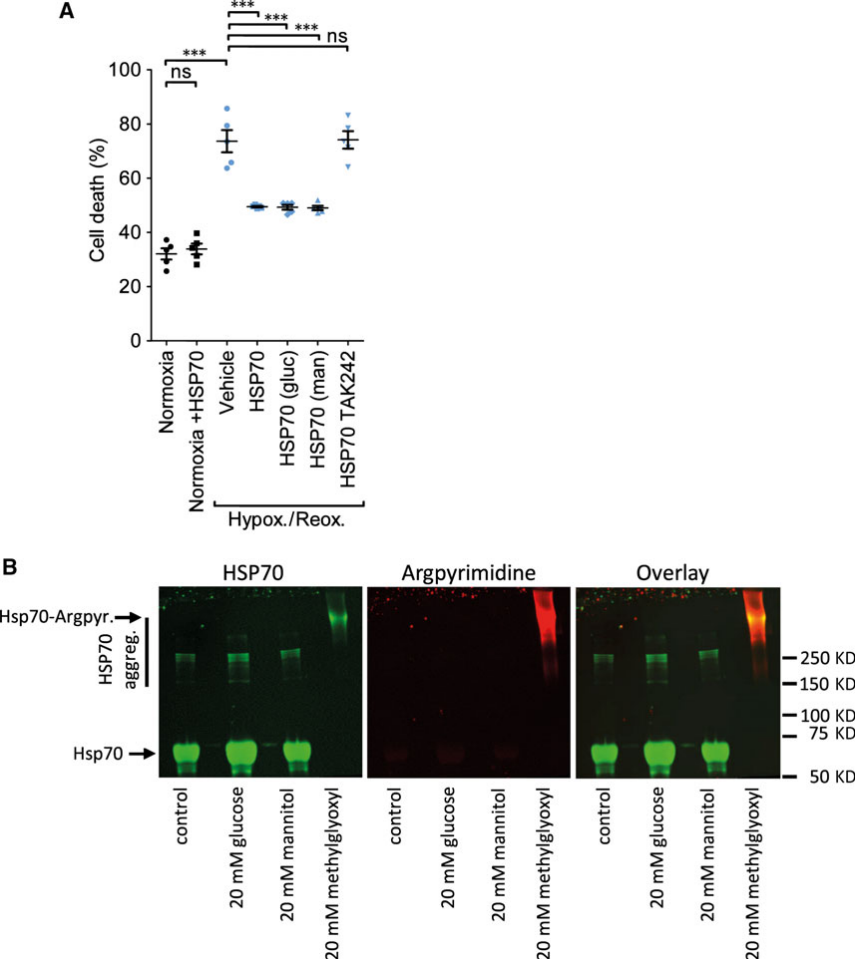
Altered levels of exosomal HSP70 or its glycation do not appear to explain the loss of cardioprotection. (**A**) Cardiomyocytes were treated with 1 ng/ml HSP70 that had been incubated 7 days in 20 mM glucose or 20 mM mannitol, prior to exposure to hypoxia and reoxygenation injury. Cell death was assessed by propidium iodide staining. TAK242 is an inhibitor of TLR4 signalling. (**B**) Western blot analysis of HSP70 treated as indicated, and probed using antibodies against HSP70 or the glycation end‐product, argpyrimidine. *N* = 4 independent experiments. Results were compared by repeated measures anova. ***P < 0.001; ns = non‐significant.

We previously demonstrated that HSP70 localized on the surface of exosomes can induce cardioprotection by signalling to TLR4 9. Hyperglycaemic conditions can cause the direct, non‐enzymatic glycation of proteins and modify their function, contributing to the development of diabetes 16. We, therefore, investigated the hypothesis that high glucose conditions inactivated exosomal HSP70. Since exosomes are unstable when incubated for longer than 24 hrs 17, we tested this hypothesis using pure, recombinant HSP70. We first confirmed that low concentrations of HSP70 are cardioprotective in our model (Figs 5A, S5). Protection was determined to be optimal at 1 ng/ml (data not shown). In accordance with our previously identified pathway of protection, TAK242, an inhibitor of TLR4 signalling, blocked protection (Fig. 5A). Pre‐incubation of HSP70 in 20 mM glucose for 7 days did not affect its ability to protect the cells against hypoxia and reoxygenation (Fig. 5A). A sample treated with mannitol was included as a non‐reactive control. Modification of arginine to argpyrimidine is one of the most common products of protein glycation, and HSP70 is susceptible to argpyrimidine modification in certain conditions 18. However, in our hand, no argpyrimidine could be detected by Western blot analysis of HSP70 after incubation with 20 mM glucose (Fig. 5B). In contrast, in a control sample of HSP70 incubated with 20 mM methyl glyoxyl, all of the HSP70 was modified and it apparently aggregated, resulting in a diffuse and slowly migrating band near the top of the gel (Fig. 5B).

**Figure 6:**
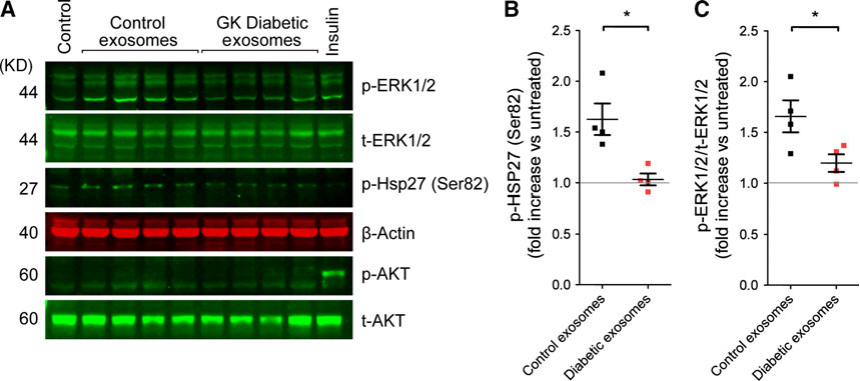
Phosphorylation of ERK1/2 and of HSP27 was significantly increased in rat cardiomyocytes treated for 5 min. with 10^8^/ml exosomes from non‐diabetic rats, but not with those treated with exosomes from GK rats (*N* = 4) (**A**–**C**). Fold increase was compared by *t*‐test. *P < 0.05.

To determine whether diabetic exosomes are capable of activating cardioprotective kinase pathways, primary cardiomyocytes were treated with exosomes purified from non‐diabetic or diabetic rats for 5 min. before harvesting and measuring kinase phosphorylation by Western blot analysis. In contrast to the significant increase in Erk1/2 and Hsp27 phosphorylation induced by non‐diabetic exosomes, no increase in phosphorylation of either protein was detected after treatment with exosomes from GK rats (*N* = 4; Fig 6A–C).

**Figure 7:**
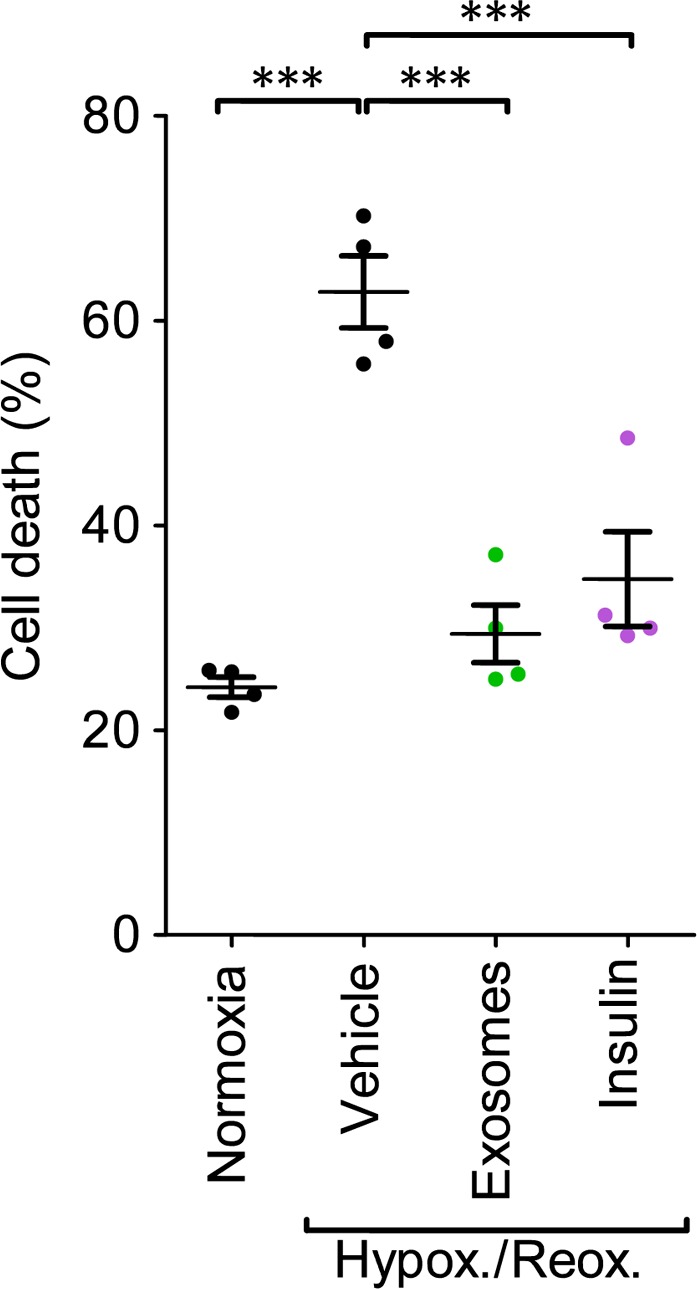
Exosomes from non‐diabetic rats protect primary adult cardiomyocytes isolated from GK diabetic rats from death after hypoxia and reoxygenation. Cardiomyocytes were treated with 10^8^/ml exosomes from GK rats. Insulin was used as a positive control. *N* = 4 independent experiments. Results were compared by repeated measures anova. ***P < 0.001.

Finally, it was important to establish whether exosomes from a healthy individual could be used to protect cardiomyocytes coming from a diabetic individual. To this end, we isolated cardiomyocytes from adult GK rats. Exposure to hypoxia and reoxygenation significantly increased cell death from 24 ± 1% to 63 ± 4% (*P* < 0.001; Figs 7, S6). Pre‐treatment with exosomes that had been isolated from control rats without diabetes significantly reduced cell death to 29 ± 3% (*P* < 0.001), which was a similar degree of protection as the positive control of insulin, which reduced death to 35 ± 5% (*P* < 0.001; *N* = 4; Fig. 7).

## Discussion

Exosomes have been suggested to mediate some of the beneficial paracrine effects of stem cells in protecting the heart against IR injury and have also been proposed as potential cardioprotective agents in their own right 5, 19. However, this property does not appear to be restricted to exosomes from stem cells, since, as we previously demonstrated, purified plasma exosomes are able to protect primary cardiomyocytes, isolated perfused rat hearts and the heart *in vivo* against IR injury 9. Similarly, exosomes secreted by cardiomyocytes overexpressing HSP20 appear to improve cardiac function and angiogenesis in diabetic mice 20. Here, we demonstrate that the ability of exosomes to activate a cardioprotective signalling pathway is impaired by diabetes in both rats and humans. Hyperglycaemia appears to be important in this effect as endothelial cells cultured in high glucose produced exosomes that were no longer cardioprotective. Despite their being significantly more HSP70 on exosomes from diabetic rats compared to non‐diabetic rats, only exosomes from non‐diabetic rats were capable of signalling to ERK1/2 and HSP27. We were unable to determine the reason for loss of protection by diabetic exosomes, but glycation of HSP70 is unlikely to be involved. Most interestingly, in terms of developing exosomes as a potential therapeutic agent, exosomes from a non‐diabetic rat retained the ability to protect cardiomyocytes from a diabetic rat.

### Why are exosomes from control blood protective?

The physiological significance of the observation that endogenous exosomes from unstimulated rats or humans are cardioprotective remains unclear. Importantly, the observation that only exosomes from non‐diabetic rats or humans are protective, greatly strengthens the interpretation that protection is not an artefact of their isolation and purification. We speculate that exosomes exert a continual ‘tonic’ cardioprotective stimulus on the heart that may be modified in response to stress. Indeed, application of an ischaemic preconditioning stimulus to the limb (‘remote ischaemic preconditioning’, or RIPC) increases the number of exosomes or extracellular vesicles in the blood of humans and rats 9, 21. However, the extent to which this increase in exosome number actually mediates the cardioprotective benefits of RIPC *in vivo* remains unclear 9, 19, 21. On the other hand, if exosomes do contribute to RIPC, then our data would suggest that defective exosomal signalling might contribute to the relative difficulty of protecting the diabetic heart using RIPC 22, 23. Future work will investigate the contribution of different blood and vascular cell types (inflammatory cells, platelets, endothelial cells, erythrocytes, etc) to the protection observed with plasma exosomes).

### What is the effect of diabetes on exosomes?

Diabetic conditions did not significantly alter the diameter of exosomes, according to our measurements by NTA and electron microscopy. Interestingly, however, there was a consistent trend towards increased diameter in both rat and human diabetic exosomes when analysed by electron microscopy. As, in contrast to NTA, exosomes must be dried for this type of analysis, it is possible this reflects an underlying difference in lipid structure and ‘collapsibility’, but this is highly speculative at this stage.

The significant increase in exosome marker proteins CD81 and HSP70 in exosomes from diabetic rats suggests an overall increase in exosome quantity in the setting of diabetes. Such an increase was not detected by NTA, which may reflect the relatively low precision of this method. In our hands, we have found that concentration determined by NTA has a relatively high coefficient of variation of ~10%, which matches published studies 24. In comparison, ELISA‐based methods are typically more precise. Despite the possible increase in exosome quantity in the blood, the diabetic exosomes were not protective, suggesting they have been modified or damaged in some way. Furthermore, despite the increase in total exosomal HSP70, the cmHsp70.1 epitope of HSP70 was not elevated, suggesting that the epitope may have been masked, modified or damaged to some extent. A recent publication demonstrated that gestational diabetes mellitus stimulates an increase in the release of exosomes from the placenta during gestation 25. The exosomes were found to significantly increase the release of proinflammatory cytokines from endothelial cells 25.

### Why does high glucose cause loss of protection?

Cardiomyocytes respond rapidly to the interaction of exosomal HSP70 with sarcolemmal TLR4 by activating the ERK1/2 cardioprotective pathway, and phosphorylation of HSP27 9. Here, we found that exosomes from diabetic rats no longer activated this pathway *in vitro*. Glucose and other sugars can non‐enzymatically modify proteins covalently by a process of glycation, and this reaction is accelerated under conditions of hyperglycaemia, as is present in diabetic blood 16. Such glycation can inactivate proteins and contributes to diabetic complications including retinopathy, nephropathy, neuropathy and arterial abnormalities 16. HSP70 has previously been identified as being susceptible to glycation, and this can cause a loss of its protein chaperone enzymatic activity 26. However, in our experiments, *in vitro* exposure to high glucose did not cause a detectable change in glycation of HSP70. It remains possible that hyperglycaemia affects other aspects of exosomal structure or function yet to be determined. Given our results, it is possible that diabetes and hyperglycaemia could also impair other signalling roles that have been ascribed to exosomes in various tissues and the vasculature 8, 27.

Surprisingly, we were not able to detect HSP70 on HUVEC exosomes by immunoassay. This would appear to indicate that HUVEC exosome induce protection by a separate mechanism, but one that is also impaired by hyperglycaemia. Interestingly, brief culture in high glucose conditions has also been shown to impair the ability of apoptotic microvesicles released from human endothelial cells to repair endothelium when administered to mice 28. In this case, the mechanism was reported to involve a decrease in exosomal miR‐126 content 28. Exosomes from various sources have also been shown to stimulate angiogenesis. On the other hand, exosomes from GK rat cardiomyocytes were found to inhibit the proliferation, migration and tube formation of a cardiac endothelial cell line 29. This was reportedly *via* a mechanism involving exosomal transfer of miR‐320, although lower miR‐126 levels were also detected in this study 29. In contrast to endothelial cells, cardiomyocytes take up relatively few exosomes 9. Furthermore, a 30 min. exposure to exosomes is far too short for sufficient miRNA to enter cells and potentially affect protein levels significantly. These factors make a role for exosomal miRNA in loss of protection of cardiomyocytes unlikely.

### Protection of diabetic hearts using non‐diabetic exosomes

Our experiments demonstrate that cardioprotective signalling can be activated in cardiomyocytes from diabetic rats using exosomes from non‐diabetic animals. A limitation of these studies is that we have not demonstrated that non‐diabetic exosomes are capable of protecting the hearts of diabetic rats when administered acutely *in vivo*. Additionally, other extracellular vesicles such as microvesicles can potentially co‐purify with exosomes isolated by differential centrifugation, and these may mediate some of the protective effect. However, our data support the idea that exosomes harvested from blood plasma or a suitable cellular source (such as the endothelial cells used here, stem cells 6, or cultured cardiomyocytes overexpressing HSP20 20 as proposed by others 30), may therefore represent a cardioprotective agent with the novel and desirable property that it is effective in the setting of diabetes 31. Important questions remain before clinical translation can be attempted, including the pharmacodynamics and pharmacokinetics of exosomes *in vivo*. Another important challenge will be the development of robust techniques for large‐scale purification of GMP‐quality, validated exosomes 7.

## Author contributions

J.A.R., K.T., J.M.V., C.B.K., V.K., C.D. and D.R. performed experiments and analysed the data. S.M.D. and D.M.Y. designed the research study, analysed the data and wrote the manuscript. All authors discussed the data and helped to revise the manuscript. We are grateful for Mark Turmaine for help with electron microscopy, Prof Gabriele Multhoff for providing the cmHsp70.1 antibody, to the volunteers who donated blood and to Dr Ashraf Hamarneh for phlebotomy.

## Conflicts of interest

The authors confirm that there are no conflicts of interest.

## Supporting information


**Figure S1** The number of exosome particles per μg protein in samples from non‐diabetic and diabetic GK rats.
**Figure S2** Representative images of normoxic cardiomyocytes, and cardiomyocytes treated with vehicle, exosomes from GK or non‐diabetic rats, or insulin, before hypoxia and reoxygenation.
**Figure S3** Representative images of cardiomyocytes treated with vehicle, exosomes from diabetic or non‐diabetic humans, before hypoxia and reoxygenation.
**Figure S4** Representative images of normoxic cardiomyocytes, and cardiomyocytes treated with exosomes from HUVEC in normoxic or high glucose conditions before hypoxia and reoxygenation.
**Figure S5** Representative images of normoxic cardiomyocytes treated with recombinant HSP70 and cardiomyocytes treated with recombinant HSP70 or insulin before hypoxia and reoxygenation.
**Figure S6** Representative images of normoxic GK cardiomyocytes, and GK cardiomyocytes treated with exosomes insulin before hypoxia and reoxygenation.
