# Supplementary material for: Cardioprotection mediated by exosomes is impaired in the setting of type II diabetes but can be rescued by the use of non‐diabetic exosomes in vitro
Source: J Cell Mol Med. 2017 Aug 25;22(1):141–51. doi: 10.1111/jcmm.13302 (PMC5742744; doi:10.1111/jcmm.13302)
Supplement: Supplementary file 1 — Figure S1 The number of exosome particles per μg protein in samples from non‐diabetic and diabetic GK rats. Figure S2 Representative images of normoxic cardiomyocytes, and cardiomyocytes treated with vehicle, exosomes from GK or non‐diabetic rats, or insulin, before hypoxia and reoxygenation. Figure S3 Representative images of cardiomyocytes treated with vehicle, exosomes from diabetic or non‐diabetic humans, before hypoxia and reoxygenation. Figure S4 Representative images of normoxic cardiomyocytes, and cardiomyocytes treated with exosomes from HUVEC in normoxic or high glucose conditions before hypoxia and reoxygenation. Figure S5 Representative images of normoxic cardiomyocytes treated with recombinant HSP70 and cardiomyocytes treated with recombinant HSP70 or insulin before hypoxia and reoxygenation. Figure S6 Representative images of normoxic GK cardiomyocytes, and GK cardiomyocytes treated with exosomes insulin before hypoxia and reoxygenation. [file JCMM-22-141-s001.pdf]

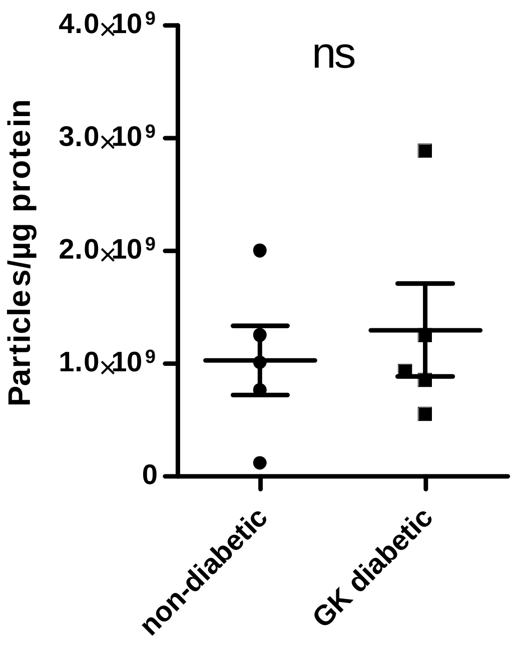

Supplementary Fig 1.
The number of exosome particles per µg protein in samples from non-diabetic and diabetic GK rats.

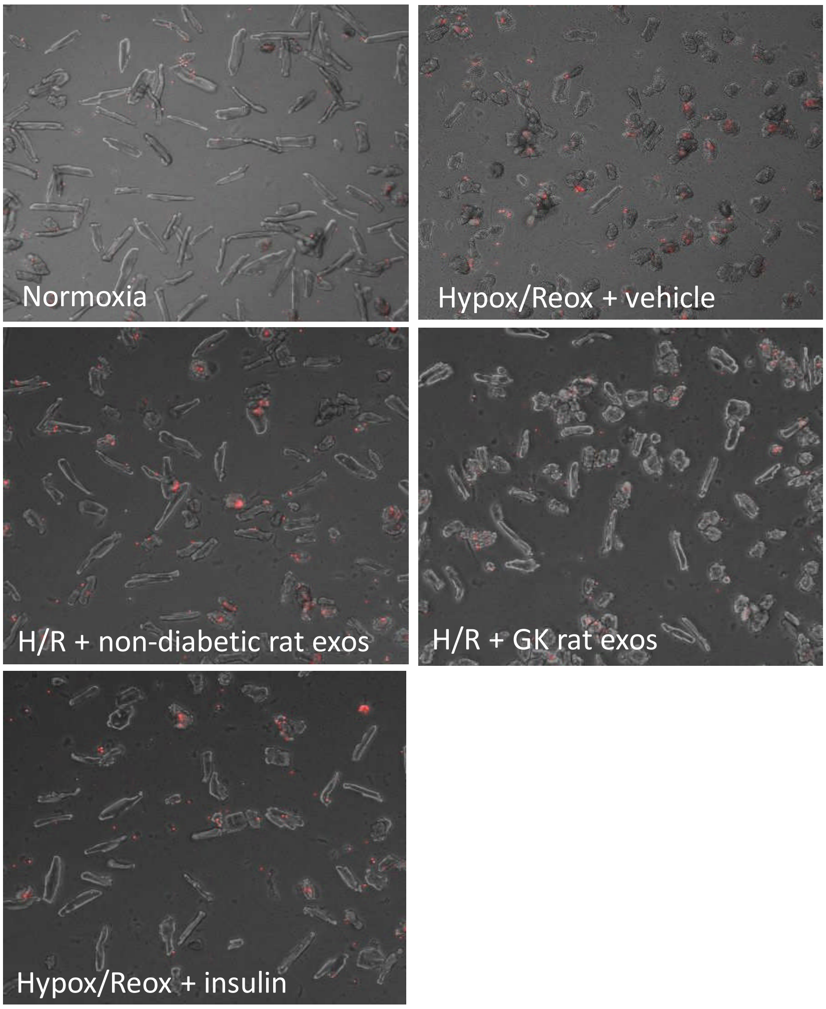

Supplementary Fig 2.
Representative images of normoxic cardiomyocytes, and cardiomyocytes treated with vehicle, exosomes from GK or non-diabetic rats, or insulin, before hypoxia and reoxygenation

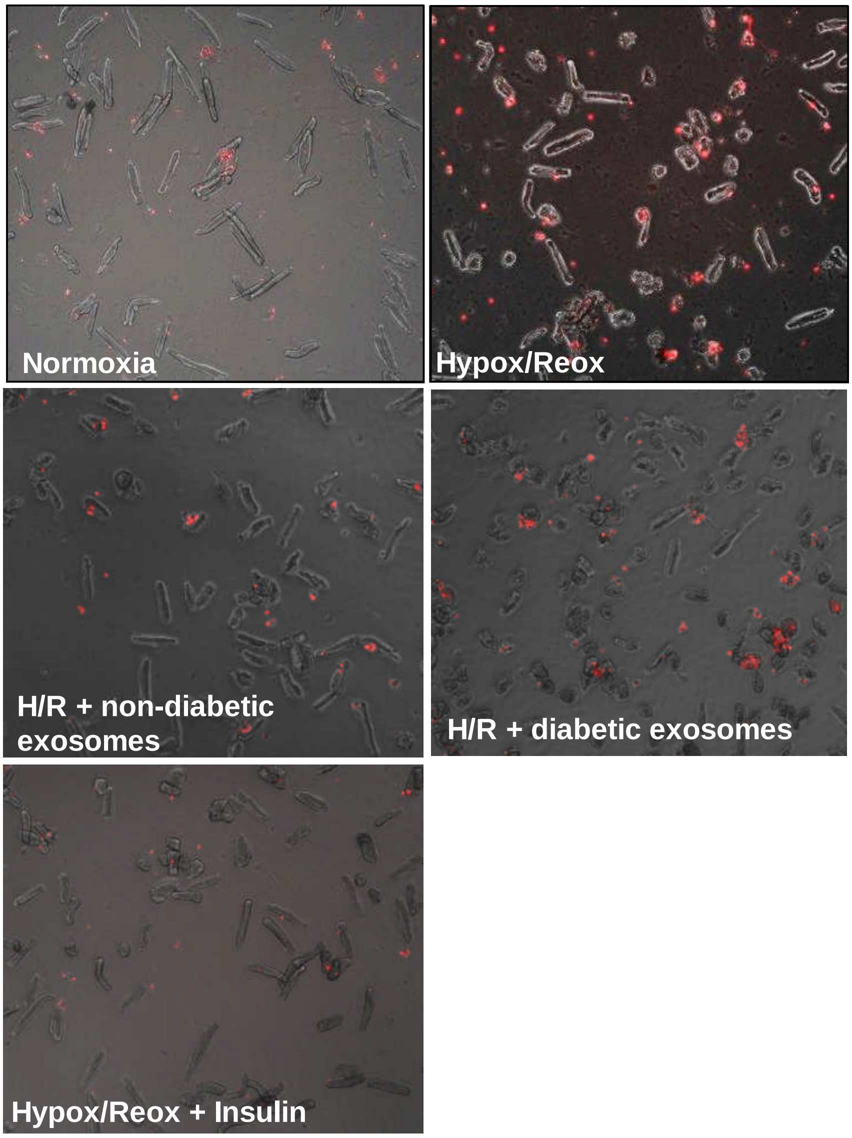

Supplementary Fig 3.
Representative images of cardiomyocytes treated with vehicle, exosomes from diabetic or non-diabetic humans, before hypoxia and reoxygenation

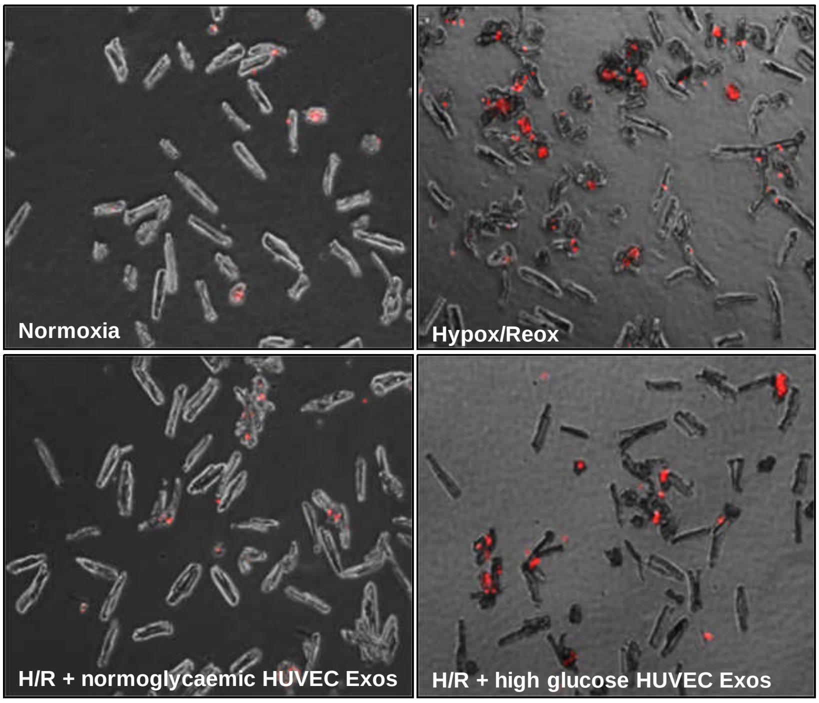

Supplementary Fig 4.
Representative images of normoxic cardiomyocytes, and cardiomyocytes treated with exosomes from HUVEC in normoxic or high glucose conditions before hypoxia and reoxygenation

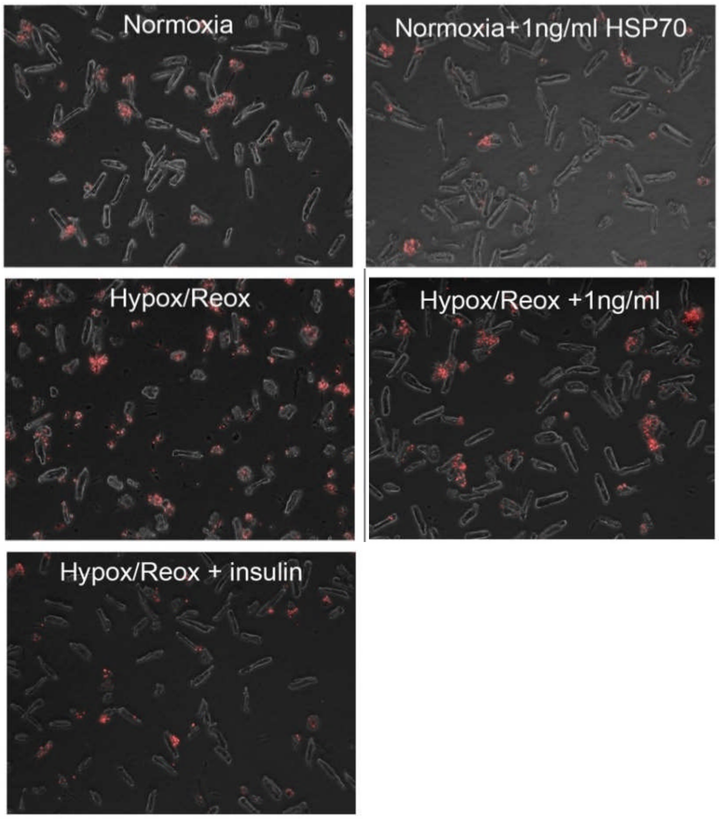

Supplementary Fig 5.
Representative images of normoxic cardiomyocytes treated with recombinant HSP70 and cardiomyocytes treated with recombinant HSP70 or insulin before hypoxia and reoxygenation

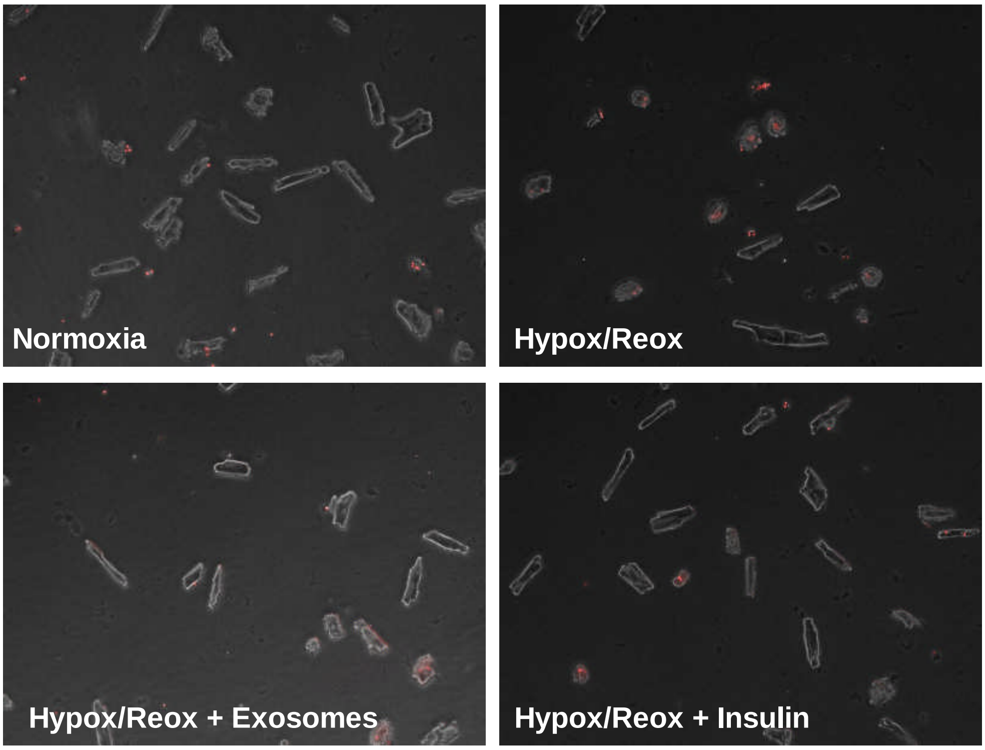

Supplementary Fig 6.
Representative images of normoxic GK cardiomyocytes, and GK cardiomyocytes treated with exosomes insulin before hypoxia and reoxygenation
